# NK92 Expressing Anti-BCMA CAR and Secreted TRAIL for the Treatment of Multiple Myeloma: Preliminary In Vitro Assessment

**DOI:** 10.3390/cells12232748

**Published:** 2023-11-30

**Authors:** Benjamin Motais, Sandra Charvátová, Zuzana Walek, Roman Hájek, Juli R. Bagó

**Affiliations:** 1 Department of Haematooncology, Faculty of Medicine, University of Ostrava, 703 00 Ostrava, Czech Republic; motais.benjamin@gmail.com (B.M.); sandra.charvatova@fno.cz (S.C.); zuzana.walek@gmail.com (Z.W.); roman.hajek@fno.cz (R.H.); 2 Faculty of Science, University of Ostrava, 701 00 Ostrava, Czech Republic; 3 Department of Haematooncology, University Hospital Ostrava, 708 00 Ostrava, Czech Republic

**Keywords:** cancer, multiple myeloma, natural killer, immunotherapy, chimeric antigen receptor, allogenic

## Abstract

Multiple myeloma (MM) has witnessed improved patient outcomes through advancements in therapeutic approaches. Notably, allogeneic stem cell transplantation, proteasome inhibitors, immunomodulatory drugs, and monoclonal antibodies have contributed to enhanced quality of life. Recently, a promising avenue has emerged with chimeric antigen receptor (CAR) T cells targeting B-cell maturation antigen (BCMA), expressed widely on MM cells. To mitigate risks associated with allogenic T cells, we investigated the potential of BCMA CAR expression in natural killer cells (NKs), known for potent cytotoxicity and minimal side effects. Using the NK-92 cell line, we co-expressed BCMA CAR and soluble tumor necrosis factor-related apoptosis-inducing ligand (sTRAIL) employing the piggyBac transposon system. Engineered NK cells (CAR-NK-92-TRAIL) demonstrated robust cytotoxicity against a panel of MM cell lines and primary patient samples, outperforming unmodified NK-92 cells with a mean difference in viability of 45.1% (±26.1%, depending on the target cell line). Combination therapy was explored with the proteasome inhibitor bortezomib (BZ) and γ-secretase inhibitors (GSIs), leading to a significant synergistic effect in combination with CAR-NK-92-TRAIL cells. This synergy was evident in cytotoxicity assays where a notable decrease in MM cell viability was observed in combinatorial therapy compared to single treatment. In summary, our study demonstrates the therapeutic potential of the CAR-NK-92-TRAIL cells for the treatment of MM. The synergistic impact of combining these engineered NK cells with BZ and GSI supports further development of allogeneic CAR-based products for effective MM therapy.

## 1. Introduction

Multiple myeloma (MM) is a hematologic malignancy affecting plasma cells (PCs) originated in the bone marrow. After non-Hodgkin lymphoma, MM is the second most common hematologic cancer and affects mostly men and older people, with a median diagnosis age of 65 years. It results from either monoclonal gammopathy of undetermined significance (MGUS) or smoldering multiple myeloma (SMM), with an increased risk over age. Clinical signs of MM can be summarized under the CRAB acronym, encompassing high serum calcium concentration, renal insufficiency, anemia, and bone lesions [1]. MM has always harbored the reputation of being incurable. Yet, the life quality of patients has significantly improved over the last decades. As per the latest data from the American Society of Cancer, the 5-year survival rate for cancer patients stands at a promising 56%. Early diagnosis plays a pivotal role in improving these outcomes, with the survival rate soaring to an encouraging 78%. However, it is essential to note that early diagnosis occurs in only a small percentage, approximately 4%, of patients. Nevertheless, the chance of relapse is inevitable, and up to 20% of MM cases evolve into extramedullary disease (EMD), a more aggressive and treatment-resistant form of the disease [2].

The initial breakthrough in MM treatment heralded the introduction of proteasome inhibitors (PIs). The first-in-class Bortezomib (BZ), approved by the Food and Drug Administration (FDA) in 2003, is now widely used in the first line of therapy against MM. BZ works by inhibiting the proteasome activity within MM cells, leading to the accumulation of proteins in the endoplasmic reticulum. This accumulation triggers a cascade of events that culminate in the activation of apoptosis pathways, effectively inducing programmed cell death in MM cells. [3]. Commonly, BZ is administrated in combination with other drugs such as immunomodulatory drugs (IMiDs) and dexamethasone [4]. IMiDs encompass analogs of thalidomide (lenalidomide, pomalidomide, and iberdomide). They demonstrated anti-proliferative effects on MM and co-stimulative properties on T and NK cells in vitro, but those effects remain uncorroborated in vivo [5]. Dexamethasone (Dex), a steroid part of glucocorticoids, has proven to be effective in all phases of the treatment of MM. It binds to glucocorticoid (GC) receptors expressed on the MM cells and specifically drives their apoptosis by the BIM protein. However, only 50% of patients respond to high doses of Dex mainly because of the MM molecular heterogeneity [6]. The introduction of monoclonal antibodies (mAbs) considerably improved the treatment of MM. Unlike the previously cited therapies, mAbs belong to targeted therapies, as they recognize specific antigens through their variable chain domain [7]. NK cells possess a unique ability to specifically detect and eliminate cancer cells through a mechanism known as antibody-dependent cell-mediated cytotoxicity (ADCC) [8]. This process involves the binding of NK cell’s FcγRIII receptor (or CD16) to antibodies that have targeted the cancer cells, enabling the NK cells to precisely engage and destroy the malignant cells. Current mAb treatments for MM involve elotuzumab, rituximab, and daratumumab, targeting SLAMF7, CD20, and CD38, respectively [9].

Over the past decade, significant strides in mAb-based therapies have paved the way for a groundbreaking technology known as chimeric antigen receptors (CARs). This approach involves the artificial fusion of the variable domain of antibodies, crucial for antigen recognition, with the T cell receptor, responsible for T cell activation [10]. This revolutionary design empowers T cells to undergo activation directly through CAR-antigen binding, eliminating the need for involvement of antigen-presenting cells, such as dendritic cells. In recent times, CAR T cells have emerged as the most promising technology in the quest to cure previously deemed incurable cancers, including MM. The remarkable success of CAR T cell therapies has been exemplified by numerous FDA approvals [11], signifying a turning point in the landscape of cancer treatment. With the implementation of more intracellular co-stimulatory domains, new generations of CAR T cells get more and more specific and powerful [12]. Most clinical trials involving CAR T cells for MM treatment target the tumor necrosis factor receptor (TNFRSF17) B-cell maturation antigen (BCMA), as it is universally overexpressed on plasma cells, but others involve other targets such as CD19, SLAMF7, and CD38 [13]. In 2021, the first anti-BCMA CAR T cell product, idecabtagene vicleucel (or Abecma), was approved by the FDA for the treatment of MM [14].

If CAR T cells are prowess to cure any cancer, many limitations are associated with their use. Indeed, the major drawbacks associated with CAR T cell use are the risks of graft-versus-host disease (GvHD) [15] and cytokine release syndrome (CRS) [16]. GvHD occurs in allogeneic transplantations due to the human leukocyte antigen (HLA) mismatch between patient and donor. Since GvHD leads to severe or even fatal events in allogeneic CAR T cell transplantation, using an autologous source of cells is preferred, requiring a personalize approach for each patient that leads to a costly process [17]. Moreover, the CRS problem is inherent with the infusion of T cells, as they get overactivated and secrete a large spectrum of cytokines, such as interleukin-6, IL-2, IL-8, IL-10, IFN-γ, and TNF-α, resulting in tissue damage [18]. Extensive gene editing of T cells can circumvent these adverse effects, such as TCR (T cell receptor) depletion to avoid GvHD [19] or suicide genes to eliminate cells when CRS is detected [20].

These challenges could potentially be addressed by employing NK cells as a platform for CAR delivery, leveraging their inherently safer characteristics. The NK cells account for 2 to 31 percent of peripheral blood lymphocytes and possess innate cytotoxic abilities against pathogens [21]. They act as the first barrier against bacterial and viral infections and can quickly detect and tackle cancer cells. To do so, they can identify a foreign or missing class-I major histocompatibility complex (MHC-I) on their target [22], leading to their activation and release of perforins that create pores in the target’s membrane and granzymes which induce apoptosis by cleaving pro-apoptotic molecules [23]. NK cell-based therapies offer a compelling advantage over CAR-T cell therapies by effectively addressing their drawbacks. Remarkably, NK cells can be infused in allogeneic conditions, representing a significant breakthrough in immunotherapy. This advantage stems from the fact that NK cells lack the TCR, which, in T cells, can inadvertently trigger recognition and attack of healthy tissues. Moreover, NK cells secrete a distinct panel of cytokines [24], contributing to a more controlled and balanced immune response and, consequently, lower risks of GvHD and CRS. In addition, persistence after the infusion is significantly lower than CAR T cells, minimizing the risk of creating long-term adverse risks [25].

Recognizing the potential of combinatorial therapy to achieve optimal outcomes, we took a comprehensive approach by engineering NK cells with a humanized nanobody-based anti-BCMA CAR [26] and the soluble form of the tumor necrosis factor-related apoptosis-inducing ligand (sTRAIL) (Appendix A). TRAIL, a naturally occurring molecule on the surface of NK cells, plays a pivotal role in combatting MM cells. When TRAIL binds to the TRAIL receptor (DR5, TRAIL-R2) overexpressed in cancer cells, it initiates a crucial programmed cell death process, which is essential for effectively combating MM. The use of a secreted form of TRAIL enhances their ability to trigger apoptosis in MM cells, significantly bolstering their anti-cancer capabilities.

Moreover, our engineered anti-BCMA-CAR NK cells expressing sTRAIL demonstrate remarkable compatibility with proteasome inhibitors, which are commonly used in MM treatment. Indeed, proteasome inhibitors have been shown to sensitize cancer cells to TRAIL-induced apoptosis, further amplifying the anti-cancer effect of our NK cell therapy [27]. Additionally, our strategy incorporates the use of GSI, which prevent the shedding of BCMA from the MM cell surface. This approach enhances the effectiveness of our anti-BCMA-CAR NK cells by increasing the retention of BCMA on MM cells, allowing for improved targeting and killing of cancer cells.

By combining these cutting-edge technologies and therapeutic approaches, we aim to create a comprehensive and potent treatment regimen that maximizes the potential of NK cell-based immunotherapy against MM, ultimately offering new hope and improved outcomes for patients fighting this challenging disease.

## 2. Results

### 2.1. Generation of Anti-BCMA CAR-NK92 Cell Line Expressing sTRAIL

**Figure 1:**
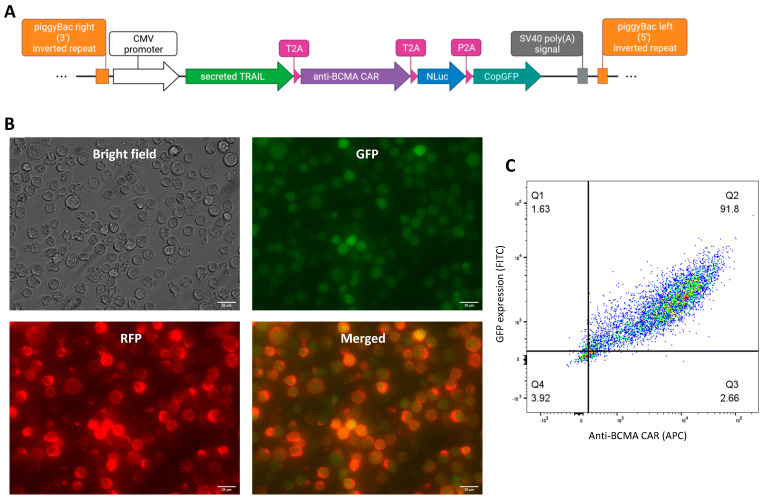
Validation of the CAR-NK92-TRAIL construct. (**A**) The expression of the anti-BCMA CAR, sTRAIL, and Nluc-GFP is driven by the CMV promoter. The coding sequences are spaced by 2A self-cleaving peptides to permit the whole expression under a unique promoter. The piggyBac LTR flanking sequences ensure the integration of the transgene in the host cell genome under the action of the piggyBac transposase. (**B**) Fluorescence microscopy image of the CAR-NK92-TRAIL cells (GFP—expressed from the transgene; RFP—conjugated to the BCMA-APC protein bound to the anti-BCMA CAR expressed at the surface of NK92 cells). Scale bar—25 μm. (**C**) Flow cytometry analysis of the GFP and anti-BCMA CAR expression by CAR-NK92-TRAIL.

The NK92 cell line was engineered by transposition of a single coding sequence encoding for our proteins of interest: the sTRAIL, the anti-BCMA CAR, and the Nluc-GFP dual reporter. The piggyBac transposon system allows, under the action of the piggyBac transposase, the integration of our plasmid DNA, flanked by inverted terminal repeat sequences, into the host’s genome via a “cut and paste” mechanism (Figure 1A). This results in the integration of multiple copies of our transgene into the NK genome, which are replicated and conserved during cell division, thus generating a stable cell line. We opted for this method as our attempts based on lentiviral approaches were infructuous, mostly due to the antiviral properties of NK cells. In our design, encoded elements were interspaced by 2A self-cleaving peptides, which ensure the generation of distinct proteins from a single mRNA, thus avoiding the use of multiple plasmids, promotors, or IRES, significantly simplifying the engineering process of our transgenic NK cell line [28]. By fluorescence microscopy, we corroborated the expression of our genes of interest [Figure 1B], with the positive green fluorescence in NK92, corresponding to the reporter gene, and the positive red fluorescence after staining the NK92 with anti-BCMA CAR antibody with APC (allophycocyanin) fluorescent reporter. Besides, the expression of the mentioned genes was quantified by flow cytometry, with more than 90% of the NK92 population positive for the expression of GFP reporter and anti-BCMA CAR, with differences in the intensity as a consequence of variations on the copy number integrated into the cell genomes [Figure 1C]. These results demonstrate the feasibility of generating a stable transgenic NK-92 cell line expressing anti-BCMA CAR, sTRAIL, and reporter proteins, without using viral vectors.

### 2.2. Phenotype and Functional Analysis of the CAR-NK92-TRAIL Cell Line

**Figure 2:**
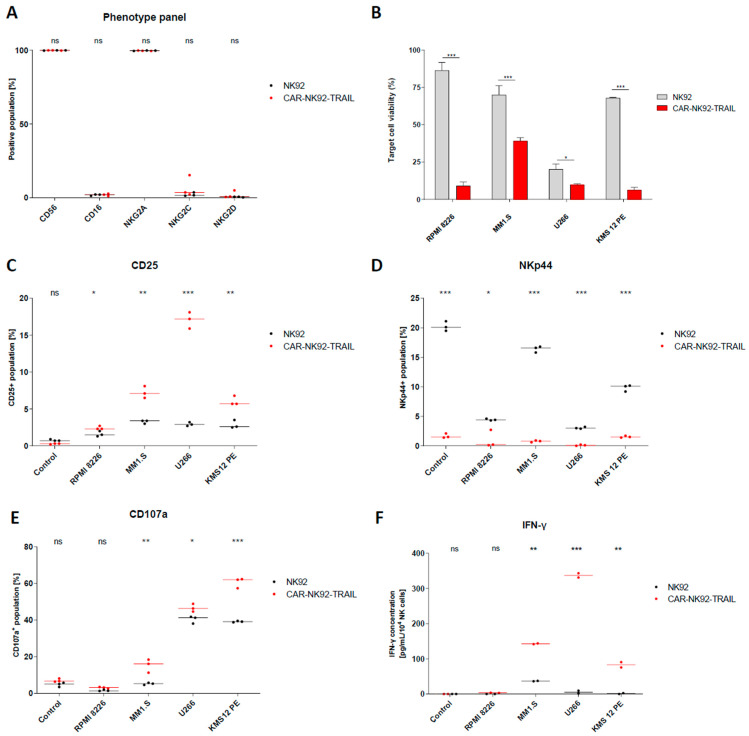
Characterization and cytotoxic effect of CAR-NK92-TRAIL against different cancer cell lines. (**A**) The expression of phenotypic markers (CD56, CD16, NKG2A, NKG2C, NKG2D) in both wt-NK92 and CAR-NK92-TRAIL cells. (**B**) Viability of the MM (RPMI-8226, MM1.S, U266, KMS-12-PE) after 24 h co-culture with wt-NK92/CAR-NK92-TRAIL cells (1:2 E:T ratio). (**C**–**E**) The expression of activation markers after 24 h co-culture with the panel of targets at (1:2 E:T ratio). Control: no target cells (**F**) Quantification of IFN-γ produced by NK cells after 24 h co-culture with the panel of targets (1:2 E:T ratio). Control: no target cells. Data are presented as means ± SD from three technical replicates. ns—not significant; * = *p* < 0.01; ** = *p* < 0.05; *** = *p* < 0.001; one-way ANOVA, repeated measures test and Student’s *t*-test.

We further evaluated the expression of NK phenotypic markers for wild-type (wt-NK92) and gene-engineered NK92 (CAR-NK92-TRAIL). Flow cytometry analysis revealed that, in both wild-type and modified NK92, the whole population is CD56+, NKG2A+, CD16-, and a minor percentage of cells express the activation markers NKG2C and NKG2D [Figure 2A]. Overall, the marker expression did not change drastically between the two populations. Next, we evaluated the ability of our CAR-NK92-TRAIL cells to mediate cytotoxic activity against our panel of cancer cell lines, consisting of four DR5+/BCMA+ MM cell lines: RPMI 8226, MM1.S, U266, and KMS-12-PE. To this end, we labeled the tumor cell lines with the constitutive expression of a dual reporter; mCherry as a fluorescent reporter for positive cell labeling, and the firefly luciferase (Fluc) as a bioluminescent reporter that permit to quantify amount of tumor cells [29]. After 24 h of co-culture with NK92 cells (E:T ratio 1:2), we observed a significantly lower number of viable MM cells in co-culture with CAR-NK92-TRAIL cells, compared to the wild-type control (wt-NK92 cells), indicating the therapeutic efficiency of the anti-BCMA CAR and the sTRAIL against MM cells [Figure 2B]. In accordance with these results, the cytotoxicity activity of wt-NK92 and CAR-NK92-TRAIL cells was associated with the expression of activation receptors [30] CD25 (IL-2 receptor) [Figure 2C] and NKp44 [Figure 2E], and the expression of molecules involved in NK cell-mediated cytotoxicity, such as degranulation marker differentiation CD107A [Figure 2D] and IFN-γ [Figure 2F]. Indeed, a remarkable increase in the expression of those markers was observed in the transgenic NK cell line, except in the case of NKp44, where a higher expression of NKp44 was observed in the wt-NK92.

### 2.3. Sensitization of MM Cells to TRAIL by BZ

**Figure 3:**
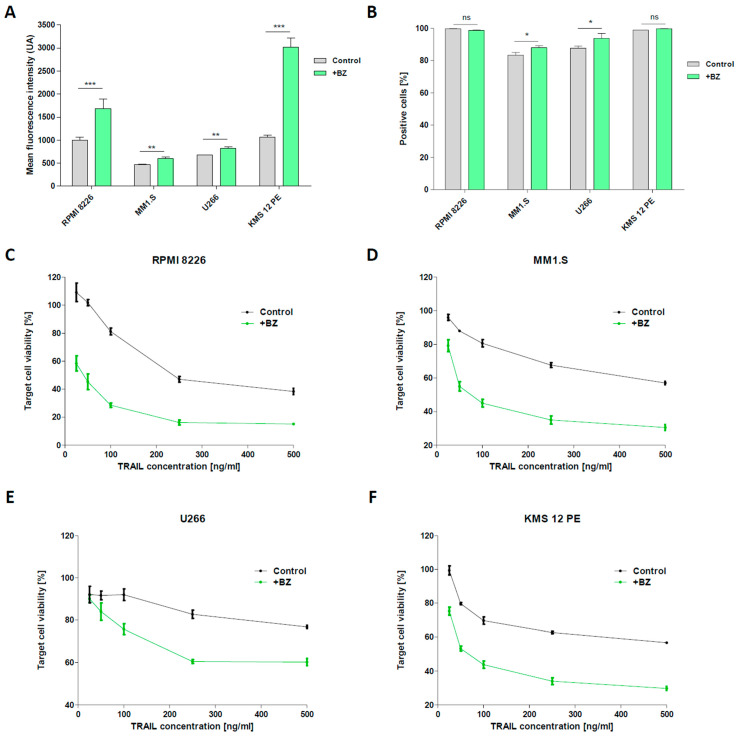
MM sensitization to TRAIL by BZ (**A,B**) DR5 (TRAIL-receptor) expression by MM cells after 24 h incubation with BZ (10 nM (MM1.S, U266) or 15 nM (RPMI-8226, KMS-12-PE) concentration). Mean fluorescence intensity (**A**) and positive populations (**B**) are indicated. (**C**–**F**) TRAIL-mediated lysis of MM cell lines after 24 h sensitization with BZ. Cells were exposed to recombinant TRAIL protein at variable concentrations and after 24 h, cell viability was assayed. Data are presented as means ± SD from three technical replicates. ns—not significant; * = *p* < 0.01; ** = *p* < 0.05; *** = *p* < 0.001; one-way ANOVA, repeated measures test and Student’s *t*-test.

In an attempt to increase the efficiency of CAR-NK92-TRAIL, we investigated several ways to sensitize MM cells to it action. The first approach revolves around the prominent use of BZ, a proteasome inhibitor commonly employed in the frontline of MM treatment [31]. MM cells express large amounts of proteins that require the proper function of their proteosome to avoid the accumulation of abnormal proteins in the endoplasmic reticulum [32]. This accumulation can lead to MM cell apoptosis. The proteasome inhibitors, such as BZ, exploit this phenomenon to reduce cell proliferation of MM cells by disrupting their proteasome. In particular, BZ targets the chymotrypsin-like subunit of the 26S proteasome, which is responsible of the degradation of ubiquitinated proteins, resulting in its inhibition. As a result, the inhibition of the proteasome causes the accumulation of ubiquitinated proteins within the cell, inducing cellular stress and ultimately prompting cell apoptosis. Previous studies also demonstrated that BZ produces the overexpression of TRAIL-receptor (DR5) [27], which once bound to the TRAIL molecule expressed on the surface of NK cells, triggers the MM cell apoptosis [33]. To verify it, we treated our panel of MM cell lines with BZ and analyzed the surface expression of DR5 by FACS. The BZ concentrations used in our study were optimized to obtain a significant increase in DR5 expression while ensuring the cells live for future assays. Even though DR5 is widely expressed on our panel of MM cell lines, ranging from about 80 to 100% of cell line populations, BZ significantly increased DR5 mean surface expression in all our cell lines [Figure 3A], and enhanced the population expression in MM1.S and U266 cell lines [Figure 3B]. We then evaluated the viability of MM cell lines when exposed to increasing concentrations of recombinant TRAIL protein, to corroborate the DR5 overexpression driven by BZ with a higher TRAIL sensitivity. We observed that when treated with BZ, the RPMI-8226, MM1.S, U266, and KMS-12-PE cell lines [Figure 3C–F] displayed higher mortality than untreated cells. The plateau of the viability curve, reached when exposed to high TRAIL concentrations, also increased after treatment with BZ. Collectively, our data confirmed the synergistic therapeutic effect of combining TRAIL with BZ.

### 2.4. Combination Treatment with GSI

**Figure 4:**
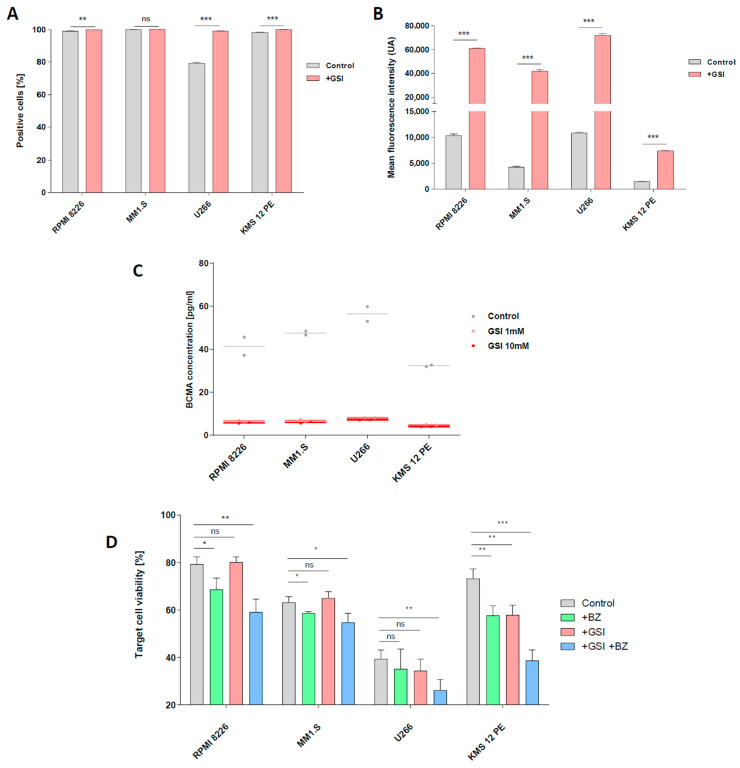
Improvement of BCMA exposure by the action of GSI and combination treatment assay (**A**,**B**) BCMA expression by MM cell lines, after 24 h incubation with γ-secretase inhibitor (GSI; 1 mM). Mean fluorescence intensity (**A**) and positive populations (**B**) are indicated. (**C**) Concentration of BCMA shed by MM cells in 24 h culture (1.5 × 10^5^ cells, 500 μL of medium) with different concentrations of GSI (0; 1 mM; 10 mM). Duplicates are represented with dots, with bars as means. (**D**) Specific lysis of MM cells in 4 h co-culture with CAR-NK92-TRAIL cells (1:1 E:T ratio). Prior assay, MM cell lines were incubated for 24 h with BZ, GSI, or BZ and GSI together. Data are presented as means ± SD from three (**A**,**B**,**D**) resp. two (**C**) technical replicates. ns—not significant; * = *p* < 0.01; ** = *p* < 0.05; *** = *p* < 0.001; one-way ANOVA, repeated measures test and Student’s *t*-test.

As BCMA is widely expressed in MM cells, it became a preferential target for CAR therapies, and many clinical trials are currently ongoing [34]. However, BCMA is known to shed from the surface of MM cells as a tumor escape mechanism, by the action of the gamma-secretase [35]. Recently, novel strategies utilize GSI to prevent the shedding of BCMA from MM cells. This action leads to the restoration of higher levels of membrane-bound BCMA in MM cells, thereby improving anti-BCMA targeted therapies [36]. Following this study, we evaluated the effect of the GSI LY3039478 using its optimal concentration (1 mM) on our panel of MM cell lines. Since our panel of MM cell lines displays a high percentage of BCMA+ cells [Figure 4A], we performed a quantitative analysis of membrane-bound BCMA by comparing mean fluorescence intensity (MFI) from fluorescent anti-BCMA antibody. We observed a clear increase in MFI in all cell lines after treatment with 1 mM GSI [Figure 4B]. The anti-shedding effect of GSI was also corroborated by the analysis of BCMA serum levels in MM cell cultures. We observed a significant reduction in BCMA in supernatants of MM cells treated with GSI. Increasing the GSI concentration to 10 mM had a slight but insignificant effect on BCMA levels, confirming previous studies [Figure 4C] [36]. To assess the therapeutic synergistic effect of the combination of BZ and GSI with our CAR-NK92-TRAIL cells, we conducted a cytotoxic assay. This assay involved pre-treating MM cells with no drug, a single drug (either BZ or GSI), or both drugs for 24 h. Subsequently, the pre-treated MM cells were washed and incubated with CAR-NK92-TRAIL cells for a duration of 4 h with an E:T ratio of 1:1. As BZ and GSI effects are reversible and gradually disappear within few hours after washing, we adjusted the experiment to reach analyzable results. Our data demonstrated that the pretreatment with BZ leads to a significant reduction in the cell viability of RPMI-8226, MM1.S, and KMS-12-PE, after cultivation with CAR-NK92-TRAIL cells. Contrarily, we did not observe a synergistic effect with U266. This data correlates with the previous results, where sTRAIL displayed an important reduction in viability in all cell lines but U266 when pretreated with BZ [Figure 4C–F]. GSI pretreatment, on the other hand, led to no significant improvement in cytotoxicity, except with the KMS-12-PE cell line. As the RPMI-8226, MM1.S, and U266 cell lines already express high levels of BCMA, despite an important shedding, they are easily detectable by the anti-BCMA CAR. On the other hand, the KMS-12-PE cell line, which displays low levels of membrane-bound BCMA, gained targetability after GSI exposure. Overall, our combinatorial treatment with both drugs and CAR-NK92-TRAIL cells, significantly increased cytotoxicity in all MM cell lines, most notably in KMS-12-PE, demonstrating a clear improvement over non-treated cells.

### 2.5. Efficiency of the Engineered CAR-NK92-TRAIL against Primary Myeloma Cells

**Figure 5:**
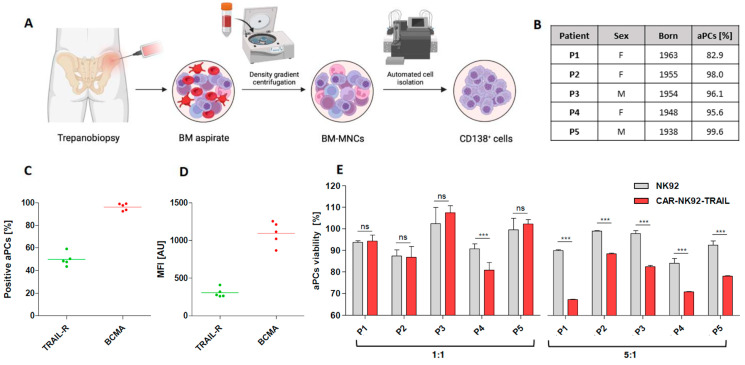
Efficiency of the engineered CAR-NK92-TRAIL cells against primary cells (**A**) Diagram depicting the obtention of primary MM cells from newly diagnosed MM patients. (**B**) Data of patients (*n* = 5) from whom primary MM cells were obtained. The percentage of aberrant plasmatic cells within the FACS-sorted CD138+ population is numbered in the “aPCs [%]” column. (**C**) Percentage of DR5+ (TRAIL-R) and BCMA+ cells within the aPC population (*n* = 5). (**D**) DR5 and BCMA mean expression by positive cells (MFI) (*n* = 5). (**E**) Cytotoxic effect of the wt-NK92 and CAR-NK92-TRAIL cells against primary MM cells (4 h assay; 1:1 and 5:1 E:T ratios). Data are presented as means ± SD from three technical replicates. ns—not significant *** = *p* < 0.001; one-way ANOVA, repeated measures test and Student’s *t*-test.

To further prove the therapeutic relevance of our CAR-NK92-TRAIL cells, we evaluated their cytotoxicity against MM cells isolated from newly diagnosed patients. BM aspirates were obtained by trepanobiopsy, and CD138+ MM cells were isolated by FACS, following Ficoll-Plaque centrifugation of mononuclear cells (Figure 5A). We first evaluated the BCMA and DR5 expression among our panel of five donors. We observed that about 50% of patient-derived aPCs expressed the DR5 marker, and 100% of them express membrane-bound BCMA [Figure 5C]. MFI analysis showed comparable TRAIL-R2/DR5 levels in all patients, while BCMA levels were more variable [Figure 5D]. Since we were not able to genetically modify aPCs with bioluminescent markers, we performed a Calcein AM-based assay to evaluate their viability after coculture with NK92 cells. This method is based on a dye that becomes fluorescent after interacting with intracellular esterases. This fluorescent Calcein is trapped within the cytoplasm until the cell membrane becomes permeable during apoptosis. By measuring the fluorescent intensity from coculture supernatants, we can quantify the percentage of dead aPCs [37]. Here, cytotoxic assays were performed at two different E:T ratios. At a 1:1 ratio, no significant differences were registered with wt-NK92 and CAR-NK92-TRAIL, except for the patient P4. However, a significant difference was observed at the E:T ratio 5:1, where CAR-NK92-TRAIL displayed a stronger effect than wt-NK92 [Figure 5E]. These data strongly corroborate previous results in MM cell lines and confirm the translational potential of our CAR-NK92-TRAIL cells.

## 3. Discussion

This past decade, novel immunotherapies changed the landscape of anti-cancer therapies, and significantly improved the life of patients [38,39]. The emergence of monoclonal antibodies [40] and subsequent advancements in CAR-T therapies [41] have revolutionized cancer treatment by providing powerful tools to precisely target and combat cancers that overexpress specific antigens. These groundbreaking therapeutic approaches have opened up new hope for patients, offering improved outcomes and enhanced possibilities for overcoming oncological challenges. Although CAR-T cells have demonstrated remarkable efficacy across a wide range of cancers, their application is not without challenges. The use of autologous CAR-T cells offers the advantage of minimizing adverse events, such as GvHD and CRS by using a patient’s own immune cells. However, this approach necessitates personalize extensive ex vivo expansion and engineering, resulting in a prolonged and costly process [18]. To overcome these limitations, researchers are exploring the concept of developing “off-the-shelf” and “universal” CAR-T cells through the depletion of TCR [42] and B2M (β-2 macroglobulin) [43]. Although is a promising “off-the-shelf” approach, it requires a substantial cell engineering and posterior cell selection. Therefore, in our study, we generated an allogenic CAR-NK cell product, which does not require considerable cell engineering and capable of circumvent limitations in CAR-T therapies [44].

Our CAR-NK approach is built upon the NK92 cell line, which stands out as the most extensively used cell line in CAR-NK clinical trials due to its ability to preserve the natural anti-cancer properties of NK cells [45,46]. We made a deliberate decision to explore this cell line for several compelling reasons. Firstly, NK92 cells exhibit the remarkable ability to proliferate indefinitely, making them an ideal foundation for generating a stable and readily available “off-the-shelf” CAR-NK cell line at a cost-effective scale. Furthermore, the process of gene-engineering NK92 cells is significantly more straightforward compared to primary NK cells. The integration of a transgene into primary NK cells poses considerable challenges, mainly attributed to their resilience against viral infection and plasmid electroporation. Recent progress was achieved in viral infection of NK cells for therapy purposes, thanks to the combination with helper molecules [47] or modifications in virus formulation [48]. Still, the use of viral vectors in therapeutic approaches is strictly framed, whereas transposable elements are more compliant with good manufacturing practice (GMP) [49]. Moreover, considering the limited ex vivo culture and post-infusion lifespan of primary NK cells, pursuing such genetic modifications becomes impractical and irrelevant. By harnessing the unique advantages of the NK92 cell line, we are positioned to develop a highly efficient and translational approach.

In our pursuit of creating a clinical-grade product, ensuring the utmost compliance with GMP standards was a priority. To this end, we designed our NK cell process expansion prioritizing safety and efficacy. While several methods for NK cell expansion involve the use of feeder cells, such as irradiated K562 engineered for the expression of membrane-bound IL-15 and 41BB ligand [50], we recognized and discarded it due to the potential safety concerns associated with their use in future clinical trials.

For this reason, we adopted a feeder-free and GMP-compliant approach for expanding our NK cells, utilizing NK MACS, an optimized medium designed for NK cell culture, supplemented with human serum, IL-2, and IL-15 [51]. To further address regulatory considerations, we employed a gene-engineering method based on the piggyBac transposon system, eliminating the need for viral vectors commonly subjected to stricter regulations [52]. By combining our host plasmid with a piggyBac transposase expression plasmid, we achieved successful integration of our target DNA into the host genome. After a few weeks, the electroporated plasmids undergo degradation, leaving no trace of bacterial origin in the NK cells, ensuring the highest level of safety and compliance [53].

In this study, we opted to express an anti-BCMA CAR to effectively target MM cells, as it is currently one of the most extensively studied antigens in clinical trials with highly promising results. Targeting BCMA enables the selective eradication of myeloma cells that overexpress the antigen, with potential off-target effects predominantly restricted to healthy BCMA-expressing plasma cells and plasmablasts, as well as a specific subset of memory B cells [54,55] The CAR we selected for our research was initially developed for CAR-T therapy, where it has demonstrated exceptional efficacy [26]. While other forms of CAR with more suitable intracellular domains (such as NKG2D, DAP10/12) have shown effectiveness in NK cells [56,57], our study establishes the legitimate use of this specific CAR in NK cells. However, cancer cells possess various resistance mechanisms that enable them to evade the action of anti-BCMA CARs. In some instances, more resistant forms of MM exhibit low or even absent levels of BCMA on their cell surface [58]. Additionally, MM cells have developed resistance mechanisms against lytic granules released by cytotoxic lymphocytes, with serpin B9 being known to inhibit the action of B granzymes, compromising CAR-mediated killing [59]. To address these challenges, our approach involves modifying our cells to secrete sTRAIL to enhance the anti-cancer capabilities of our NK cells. Although TRAIL is naturally present on the surface of our NK cells, the secreted form of TRAIL has a higher propensity to reach cancer cells and trigger apoptosis, making it an effective strategy to combat MM cells through different mechanisms. Furthermore, the utilization of TRAIL as an anti-cancer therapeutic agent has been previously evaluated in mouse models, affirming both its efficacy and its remarkable absence of off-target toxicity [60]. By employing this multi-faceted approach, our goal is to eliminate MM cells, particularly the most resistant ones, and significantly improve the therapeutic outcomes in treating this challenging and complex disease.

To assess the anti-tumor effect of the CAR-NK92-TRAIL cells, we conducted a comprehensive comparison with the wild-type NK92 cells. Our study encompassed several MM cell lines to replicate the heterogeneity seen in primary tumors [61]. Our findings revealed a significant increase in cytotoxicity when CAR-NK92-TRAIL engaged against MM cell lines, and this enhanced response correlated with the overexpression of functional markers CD25, CD107a, and IFN-γ.

To enhance the efficiency of the CAR-NK92-TRAIL cells against MM, we explored combination therapy with molecules capable of augmenting the effects of both the anti-BCMA CAR and secreted TRAIL. In this context, the action of the proteasome inhibitor BZ in sensitizing for TRAIL has been previously demonstrated [27], and our results fully corroborated these findings. The use of BZ is particularly promising as it is widely employed in the first line of MM treatment [62,63], making it an ideal complement to our CAR-NK92-TRAIL cells. Additionally, we investigated the potential of GSI, which could be effective in targeting MM cells expressing low levels of BCMA that might otherwise evade the action of anti-BCMA CAR [36]. By combining these elements, we anticipate a significant improvement in the treatment of incurable MM, offering a more comprehensive and targeted therapeutic approach.

To strengthen the evidence supporting the effectiveness of our therapeutic approach, future studies involving in vivo mouse models are essential. In these investigations, our CAR-NK92-TRAIL cells, derived from the well-established NK92 cell line, will be subjected to gamma irradiation as is performed in clinical trials to prevent its proliferation. It is noteworthy that the use of the NK92 cell line has already demonstrated clinical relevance in ongoing clinical trials [64]. This approach not only aligns with current clinical practices but also ensures the suitability of our preclinical findings for potential clinical translation.

## 4. Materials and Methods

### 4.1. Cell Culture

#### 4.1.1. Culture Conditions

NK-92 cells were cultured in non-adherent flasks with complete NK MACS medium (Miltenyi, Bergisch Gladbach, Germany) supplemented with 5% of human AB serum (Sigma-Aldrich, Munich, Germany), 500 U/mL of interleukin-2 (IL-2; Peprotech, London, UK), 100 ng/mL of interleukin-15 (IL-15; Peprotech), and 0.5% of penicillin-streptomycin (Sigma-Aldrich, Munich, Germany). The medium was changed twice a week, and cells were routinely counted using the Luna-Stem Dual Fluorescence Cell Counter (Logos Biosystems, Anyang, Korea, version 1.4.0) to maintain a concentration of 2 × 10^5^ to 5 × 10^5^ cells/mL.

Our panel of cancer cells consisted of human MM cell lines RPMI-8226 (ATCC CCL-155), MM1.S (ATCC CRL-2974), U266 (ATCC TIB-196) and KMS-12-BM (DSMZ ACC 551). RPMI-8226, MM1.S, and KMS-12-BM cell lines were cultured in the RPMI-1640 medium (Sigma-Aldrich, Munich, Germany) supplemented with 10% of FBS (Sigma-Aldrich, Munich, Germany), 1% of Ultraglutamine-1 (Lonza, Basel, Switzerland), and 1% of penicillin/streptomycin (P/S; Lonza, Basel, Switzerland). The medium was routinely changed three times a week and the cell concentration was maintained close to 5 × 10^5^ cells/mL.

#### 4.1.2. Cell Treatments

MM cell lines were seeded at 2.5 × 10^5^ cells/mL in 6-well plates in RPMI-1640 complete medium and treated with BZ at the following concentrations: 7.5 nM for RPMI-8226 and KMS-12-BM, and 5 nM for MM1.S and U266. Cells were left in culture for 24 h prior to experiments. Cells were also treated with GSI (LY3039478; SelleckChem, Planneg, Germany) for 24 h at a concentration of 1 μM.

### 4.2. Plasmid Constructs

#### 4.2.1. Lentiviral Plasmids

A lentiviral construct was designed for the constitutive expression of the red fluorescence (mCherry, mCh) and firefly luciferase (FLuc). We obtained the coding sequences by amplifying the cDNA encoding both reporters (Addgene #44965, Watertown, MA, USA) and cloned in a lentivirus backbone (Addgene #12262, Watertown, MA, USA) using standard cloning procedures.

#### 4.2.2. Expression Plasmids

The transposase plasmid was built by cloning the coding sequence of the hyperactive transposase, synthesized from a source article [65], in a CMV promotor expression plasmid. The plasmid encoding anti-BCMA CAR, sTRAIL, and Nluc-GFP was built by assembling the coding sequences of interest between the transposable elements of our destination plasmid. We first performed DNA amplification of the coding sequences from the following sources: The anti-BCMA CAR was synthesized from a source article [26], the sTRAIL coding sequence (FLT3L-TRAIL fusion protein) was obtained following a protocol established in the literature [33] and the dual reporter GFP-P2A-NLuc was amplified from the original plasmid (Addgene #73032, Watertown, MA, USA). The amplicons were designed to be all cloned by Gibson assembly into the destination plasmid interspaced with 2A self-cleaving peptides as described in the Figure 1A. The proper orientation of the insert was further assayed by restriction analysis and agarose gel electrophoresis.

### 4.3. Gene Engineering

#### 4.3.1. Lentiviral Transduction of Cancer Cells

Our lentiviral constructs were packaged in human embryonic kidney 293FT cells (HEK 293FT, a kind gift of Prof. Václav Hořejší, Institute of Molecular Genetics, Prague, Czech Republic) as lentiviral vectors. In DMEM medium (Sigma-Aldrich, Munich, Germany) with 10% of FBS without antibiotics, 293FT cells were seeded at 3–4 × 10^6^ cells per 10 cm dish. On the next day, the cells were transfected with one dual reporter plasmid (either FLuc-mCh or NLuc-GFP), the pMD2.G plasmid encoding the VSV-G envelope (Addgene #12259, Watertown, MA, USA), and the psPAX2 packaging plasmid (Addgene #12260, Watertown, MA, USA) using the JetPRIME^®^ transfection system (Polyplus, Illkirch-Graffenstaden, France). After 5 h of transfection, the medium was replaced, and after 64 h the viral supernatant was collected. Then, we removed cell contaminants using a 0.45 μm filter and concentrated the virus from the supernatant using Amicon^®^ Ultra-15 centrifugal filter unit (Merck Millipore, Tullagreen, Ireland). The different tumor cell lines were infected with the concentrated viruses at a varying multiplicity of infection in culture media containing 8 µg/mL of Polybrene (Sigma-Aldrich, Munich, Germany).

#### 4.3.2. Electroporation of NK-92

NK-92 cells were electroporated using the Neon transfection system (Thermo Scientific, Waltham, MA, USA) and following a pre-established protocol [66]. Briefly, PBNK were washed in OptiMEM medium (Sigma-Aldrich, Munich, Germany) with 0.1% of a solution of saturated cholesterol (Sigma-Aldrich, Munich, Germany) with DMSO (Panreac AppliChem, Darmstadt, Germany), and centrifuged at 100× *g* for 10 min. NKs were then resuspended in OptiMEM medium (Sigma-Aldrich, Munich, Germany) at a concentration of 4 × 10^7^ cells/mL and mixed with a plasmid solution made of the CAR-carrying and the transposase plasmids at a 1:1 ratio and final DNA concentration of 120 μg/mL. A total of 100 μL of the cell-DNA mixture was electroporated in the buffer E2, with a first pulse of 1650 V, 20 ms, and a second of 500 V, 100 ms. The cells were then poured in 400 μL of preheated RPMI basal medium (Sigma-Aldrich, Munich, Germany) on a 6-well plate and rested for 10 min, before adding 1.5 mL of complete NK MACS medium (Miltenyi, Bergisch Gladbach, Germany), without P/S. Cells were cultured and sorted for the expression of the transgenes by FACS (fluorescence-activated cell sorting).

### 4.4. Flow Cytometry Analyses

All flow cytometry assays were performed by FACS using BD FACSAria TM III Cell Sorter (BD Biosciences-US, San Jose, CA, USA, version 8.0.1). Data were gathered from 20,000 detected events, and replicates were always stained separately.

#### 4.4.1. Assessment of the Purity of CAR-NK92-TRAIL

The purity of our CAR-NK92-TRAIL product was verified by analysis of the GFP fluorescence expressed from the transgene, and for CAR expression after staining with the fluorescent human recombinant CD269/BCMA/TNFRSF17 protein, PE (R&D Systems, Minneapolis, MN, USA). MM cell lines and patient samples were assayed for the expression of TRAIL-receptor after staining with anti-TRAIL-R2 receptor (CD262) antibody, APC (Sony biotechnologies, San Jose, CA, USA), and for the expression of BCMA after staining with anti-BCMA (CD269) antibody, APC (Miltenyi, Bergisch-Gladbach, Germany).

#### 4.4.2. Phenotype Panel Analysis

NK92 cells were assayed for the expression of the following phenotypic markers of NK cells: CD56-APC-Cy7-Vio770 (Miltenyi, Bergisch Gladbach, Germany), CD16-APC (Miltenyi, Bergisch Gladbach, Germany), NKG2A-PE-Vio770 (Miltenyi, Bergisch Gladbach, Germany), NKG2C-PE (Miltenyi, Bergisch Gladbach, Germany), NKG2D-PerCP-Cy5.5 (Biolegend, San Diego, CA, USA), KIR2D-FITC (Miltenyi, Bergisch Gladbach, Germany), and SYTOX^®^ Blue dead cell stain (Invitrogen, Waltham, MA, USA) to evaluate cell viability.

#### 4.4.3. Functional Panel Analysis

The expression of three cytotoxic markers, the IL-2 receptor (CD25-VioBright-FITC (Miltenyi, Bergisch Gladbach, Germany)), the lysosome-associated membrane glycoprotein (CD107a-PE (Miltenyi, Bergisch Gladbach, Germany)), and the natural cytotoxicity-triggering receptor (NKp44-PE-Vio770 (Miltenyi, Bergisch Gladbach, Germany)) were analyzed by FACS after cultivation of 1.5 × 10^5^ effector cells (either NK92 or CAR-NK92-TRAIL) with 3 × 10^5^ cells from our MM panel (1:2 E:T ratio) for 24 h, in a 24-well plate with complete RPMI-1640 medium (Sigma-Aldrich, Munich, Germany).

### 4.5. Cytotoxic Assays

#### 4.5.1. Cytotoxic Assays on MM Cell Lines

We performed cytotoxic assays in quadruplicates in flat 96-well plates. NK-92 and CAR-NK92-TRAIL were co-cultured with 2 × 10^4^ target tumor cells (RMPI-8226, MM1.S, U266, and KMS-12-BM) at variable effector: target (E:T) ratios in 100 μL of complete RPMI-1640 medium. Cells were co-cultured for the indicated time before measuring bioluminescence (BLI) using Infinite^®^ F Plex (Tecan Männedorf, Switzerland) controlled by software i-controlTM (Tecan, Männedorf, Switzerland, version 3.9.0.1), after addition of D-luciferin potassium salt (Goldbio, St. Louis, MO, USA) to the wells at a final concentration of 0.5 mg/mL. BLI values were registered for a kinetic of 10 min and peak values were taken from each well. The cell viability percentage was measured following the equation:

$$\%\;Cell\;viability=\frac{BLI\;replicate\;sample}{mean\;BLI\;control}*100$$

#### 4.5.2. Cytotoxic Assays on Patient Samples

We performed cytotoxic assays on five samples from different patients newly diagnosed with MM. Prior to co-culture with effector cells, aPCs from patient samples were labeled with eBioscienceTM Calcein AM viability dye (Invitrogen, Waltham, MA, USA) according to the manufacturer’s protocol. Then, 2 × 10^4^ aPCs were co-cultured with either 2 × 10^4^ (E:T = 1:1) or 1 × 10^5^ (E:T = 5:1) effector cells, either NK92 or CAR-NK92-TRAIL. Assays were performed on triplicates in 24-well plates for four hours in a total volume of 500 μL. After that, plates were spinoculated (300× *g*, 5 min) and 100 μL of supernatant was collected from the wells to measure fluorescence release from dead aPCs. Fluorescence intensity (excitation 495 nm, emission 515 nm) was measured using Infinite^®^ F Plex (Tecan, Männedorf, Switzerland) controlled by software i-controlTM (Tecan, Männedorf, Switzerland, version 3.9.0.1). Cell viability (%) was evaluated based on the fluorescence intensity of Calcein released into the medium, following this equation:

$$\%\;Cell\;viability=\frac{Fluoresc.replicate\;sample}{mean\;Fluoresc.control}*100$$

### 4.6. Quantification of Protein by ELISA

#### 4.6.1. Quantification of Shed BCMA from MM Cells

To assay the concentration of BCMA they shed, 1.5 × 10^5^ of each MM cell line were seeded in duplicates with 500 mL of complete RPMI-1640 medium in 24-well plates. Cells were assayed for GSI treatments at concentrations of 1 μM and 10 μM. After 24 h, plates were spinoculated (300× *g*, 5 min) and the supernatant was collected for each condition. Samples were diluted 1:10 in sample diluent prior to ELISA, following the Human BCMA/TNFRSF17 ELISA Kit (Invitrogen, Waltham, MA, USA) instructions. Absorbance was measured on the micro-plate reader Infinite F Plex (Tecan, Männedorf, Switzerland, version 3.9.0.1) at 450 nm. BCMA concentrations were calculated based on the kit’s standard curve measured alongside the samples.

#### 4.6.2. Quantification of IFN-γ Secretion by CAR-NK92-TRAIL

CAR-NK92-TRAIL cells were assayed for the expression of IFN-γ after co-culture with our panel of MM cell lines. 7.5 × 10^4^ NKs were incubated in triplicates with 1.5 × 10^5^ target cells in 500 μL of complete RPMI medium in 24-well plates. After 24 h, plates were spinoculated (300× *g*, 5 min) and the supernatant was collected for each condition and quantified for IFN-γ concentration by IFN-γ ELISA kit (Invitrogen, Waltham, MA, USA), following the manufacturer’s protocol. Absorbance was measured on the micro-plate reader Infinite F Plex (Tecan, Männedorf, Switzerland, version 3.9.0.1) at 450 nm. IFN-γ concentrations were calculated based on the kit’s standard curve measured alongside the samples.

## 5. Conclusions

The results presented in this study demonstrate the efficiency of our novel immunotherapy against MM, which combines the anti-BCMA CAR, and sTRAIL expressed by the NK92 cell line. We highlighted its potential in a combinatorial therapy with the proteasome inhibitor BZ, and the GSI to further increase its anti-cancer properties. Future studies will focus on assessing the efficiency of CAR-NK92-TRAIL cells in vivo with mouse models of MM.

## Figures and Tables

**Figure A1:**
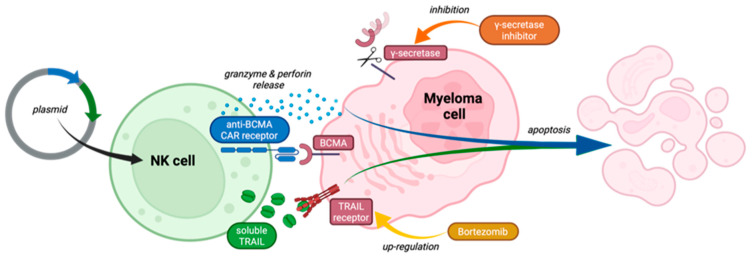
Overview of the Synergistic Combination Therapy: In our proposed approach, we have genetically modified the NK-92 cell line to express both the anti-BCMA CAR receptor and soluble TRAIL to induce apoptosis in multiple myeloma cells by targeting BCMA and the TRAIL receptor, respectively. Combination therapy with γ-secretase inhibitor and bortezomib improved the expression of the therapeutic targets on myeloma cells, significantly enhancing the killing potential of the proposed NK cell model.

## Data Availability

The data presented in this study are available from the corresponding author upon request.

## Abbreviations

(a)PCs	(aberrant) plasma cells
(c)DNA	(complementary) deoxyribonucleic acid
(s)TRAIL	(secreted) tumor necrosis factor-related apoptosis inducing ligand
ADCC	antibody-dependent cellular cytotoxicity
ANOVA	analysis of variance
APC	allophycocyanin
B2M	β2 microglobulin
BCMA	B-cell maturation antigen
BZ	bortezomib
CAR	chimeric antigen receptor
CMV	cytomegalovirus
CRS	cytokine release syndrome
DMSO	dimethyl sulfoxide
E:T	effector to target ratio
ELISA	enzyme-linked Immunosorbent Assay
EMD	extramedullary disease
FACS	fluorescence Activated Cell Sorting
FBS	fetal bovine serum
FDA	Food and Drug Administration
Fluc	firefly luciferase
GC	glucocorticoid
GFP	green fluorescent protein
GMPs	good manufacturing practices
GS(I)	gamma-secretase (inhibitor)
GvHD	graft versus host disease
HLA	human leukocyte antigen
IFN-γ	interferon gamma
IL	interleukin
IMiDs	immunomodulatory drugs
IRES	internal ribosome entry site
LTR	long terminal repeat
mAbs	monoclonal antibodies
MFI	mean fluorescence intensity
MGUS	monoclonal gammopathy of undetermined significance
MHC-I	class-I major histocompatibility complex
(S)MM	(smoldering) multiple myeloma
mRNA	(messenger) ribonucleic acid
NK	natural killer
Nluc	nanoluc luciferase
P/S	penicillin/streptomycin
PI	proteasome inhibitor
TCR	T cell receptor
TNF-α	tumor necrosis factor alpha
wt	wild-type
